# Risk factors for nonunion after intramedullary nailing of subtrochanteric femoral fractures

**DOI:** 10.1007/s00402-019-03131-9

**Published:** 2019-02-07

**Authors:** Dietmar Krappinger, Bernhard Wolf, Dietmar Dammerer, Martin Thaler, Peter Schwendinger, Richard A. Lindtner

**Affiliations:** 1 Department of Trauma Surgery, Medical University of Innsbruck, Anichstraße 35, 6020 Innsbruck, Austria; 2 Department of Orthopaedics, Medical University of Innsbruck, Anichstraße 35, 6020 Innsbruck, Austria

**Keywords:** Subtrochanteric femoral fracture, Subtrochanteric fracture, Femoral fracture, Nonunion, Pseudarthrosis, Risk factors, Intramedullary nailing, Femoral nailing

## Abstract

**Introduction:**

Nonunion is a common complication after intramedullary nailing of subtrochanteric femoral fractures. A more detailed knowledge, particularly of avoidable risk factors for subtrochanteric fracture nonunion, is thus desired to develop strategies for reducing nonunion rates. The aim of the present study therefore was to analyse a wide range of parameters as potential risk factors for nonunion after intramedullary nailing of subtrochanteric fractures.

**Materials and methods:**

Seventy-four patients who sustained a subtrochanteric fracture and were treated by femoral intramedullary nailing at a single level 1 trauma centre within a 6-year period were included in this study. A total of 15 patient-related, fracture-related, surgery-related, mechanical and biological parameters were analysed as potential risk factors for nonunion. Furthermore, the accuracy of each of these parameters to predict nonunion was calculated.

**Results:**

Nonunion occurred in 17 of 74 patients (23.0%). Of the 15 potential risk factors analysed, only 3 were found to have a significant effect on the nonunion rate (*p* < 0.05): postoperative varus malalignment, postoperative lack of medial cortical support and autodynamisation of the nail within the first 12 weeks post-surgery. Accuracy of each of these 3 parameters to predict nonunion was > 0.70. Furthermore, the nonunion rate significantly increased with the number of risk factors (no risk factor: 2.9%, one risk factor: 23.8%, two risk factors: 52.9%, and three risk factors: 100% [Chi-square test, *p* = 0.001)].

**Conclusions:**

Our study indicates that intraoperative correction of varus malalignment and restoration of the medial cortical support are the most critical factors to prevent nonunion after intramedullary nailing of subtrochanteric femoral fractures. In addition, autodynamisation of the nail within the first 3 months post-surgery is a strong predictor for failure and should result in revision surgery.

## Introduction

Subtrochanteric fractures account for approximately 5–20% of all proximal femoral fractures [1, 2]. They occur after high-energy trauma mainly in younger patients, as osteoporotic fractures in the elderly and as bisphosphonate-associated atypical fractures [3–5]. Although the use of extramedullary devices, such as sliding hip screws [6], blade plates [7] and locking compression plates [8], has been described in the literature, antegrade femoral intramedullary nailing is generally considered the gold standard for subtrochanteric fracture stabilisation [9–12].

The subtrochanteric region is an anatomical region with distinct mechanical and biological properties. Stress concentrations in the subtrochanteric region are among the highest in the entire body [13, 14]. Additionally, the subtrochanteric region is mainly composed of cortical bone with critical blood supply [8, 14, 15]. These factors may account for higher nonunion rates after internal fixation of subtrochanteric fractures compared to other anatomical regions [13, 16, 17].

Several potential risk factors for nonunion, such as varus malreduction [18], residual displacement after reduction [17], lack of medial cortical support [10], and bisphosphonate-associated fractures [4], have been described in the literature. These studies, however, have typically focused on one particular factor only. The aim of the present study, therefore, was to assess risk factors for nonunion after intramedullary nailing of subtrochanteric femoral fractures in a multivariate setup.

## Materials and methods

A consecutive series of 74 patients was included in this retrospective analysis of prospectively collected data. Subtrochanteric fractures were defined as fractures with the centre of the primary fracture line in the subtrochanteric region. The latter includes the femoral segment between the lower border of the lesser trochanter and a point 5 cm distal from this border [9, 10, 19]. Inclusion criteria were: (1) primary surgical treatment by antegrade femoral intramedullary nailing, (2) age > 18 years, and (3) surgical treatment and follow-up examinations at the same Level-1 trauma centre. Exclusion criteria were: (1) fractures resulting from primary or metastatic bone tumours, (2) periprosthetic fractures or peri-implant fractures, (3) intertrochanteric fractures with fracture extension into the subtrochanteric region, and (4) patients with incomplete radiological follow-up. Previous knee arthroplasty was not an exclusion criterion.

In all patients, surgical stabilisation was performed within 3 days after trauma with the patients positioned supine on a fracture table. Closed reduction was performed when applicable with an acceptable reduction. Otherwise the fractures were reduced openly via a lateral submuscular approach. The decision to additionally use cerclage wires was made by the surgeon on an individual basis. Three different nails (AFN / PFNA / TFN, Synthes, Oberdorf, Switzerland) were used for internal fixation and share the trochanter tip as their proximal entry point. Intramedullary reaming was performed in atypical femoral fractures [20].

Postoperative mobilization included partial or full weight bearing according to the surgeon´s advice. Full weight bearing was allowed in all patients with atypical fractures or for geriatric patients, who were not able to perform partial weight bearing. The radiological follow-up included anterior–posterior (AP) and lateral radiographs after 6 weeks, 12 weeks, 6 months and additionally 12 months if necessary. Clinically, nonunion was defined as persistent pain at the fracture site during weight bearing after 6 months [3, 10, 21]. Radiologically, nonunion was defined as lack of cortical bridging after 6 months on at least three cortices [3, 4, 12, 21].

The following parameters were analysed as potential risk factors for nonunion after intramedullary nailing of subtrochanteric fractures:

1. Patient-related parameters
  - Age
  - Gender
  - Osteoporosis
2. Fracture-related parameters
  - High-energy vs. low-energy fracture
  - Fracture type according to the Seinsheimer classification
  - Distance between the trochanter tip and the centre of the primary fracture line
3. Surgery-related parameters
  - Residual displacement in AP view
  - Residual displacement in lateral view
  - Static vs. dynamic locking of the nail
4. Mechanical parameters
  - Varus malalignment after reduction
  - Medial cortical support after reduction
  - Autodynamisation due to loosening or breakage of distal locking bolts
5. Biological parameters
  - Open reduction
  - Use of cerclage wires
  - Bisphosphonate-associated atypical fractures

**Fig. 1:**
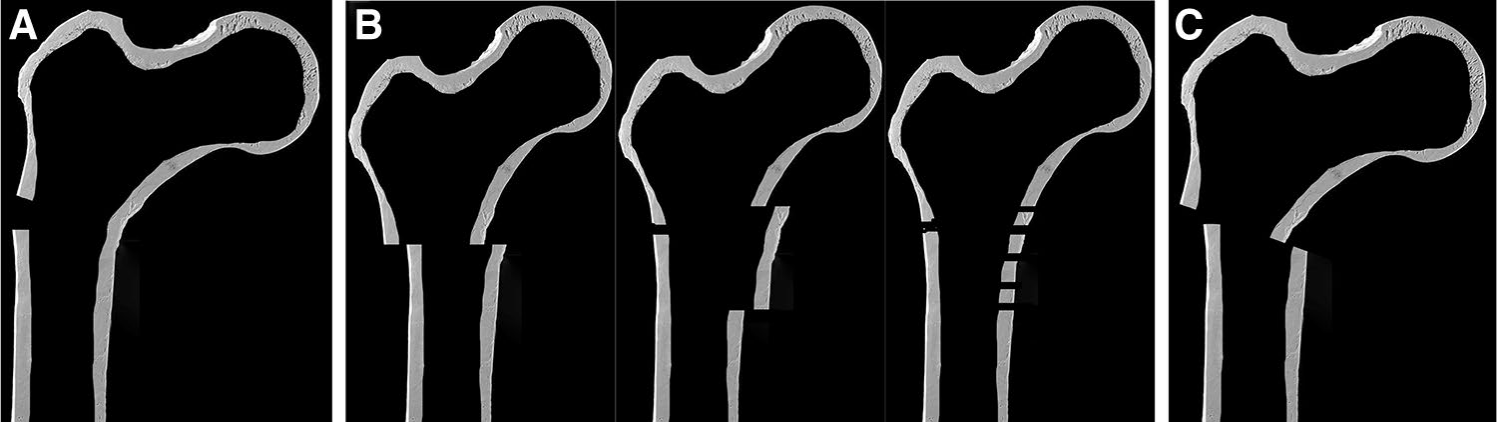
Schematic illustration of varus malalignment, lack of medial cortical support and combination of both. **a** Varus malalignment, but restored medial cortical support. **b** Lack of medial cortical support due to nonanatomic reduction (left), a large displaced medial butterfly fragment (middle) or medial comminution (right), but no varus malalignment. **c** Varus malalignment combined with lack of medial cortical support due to nonanatomic reduction (lack of medial cortical support due to medial comminution or a displaced medial fracture fragment not depicted for reasons of clarity)

**Fig. 2:**
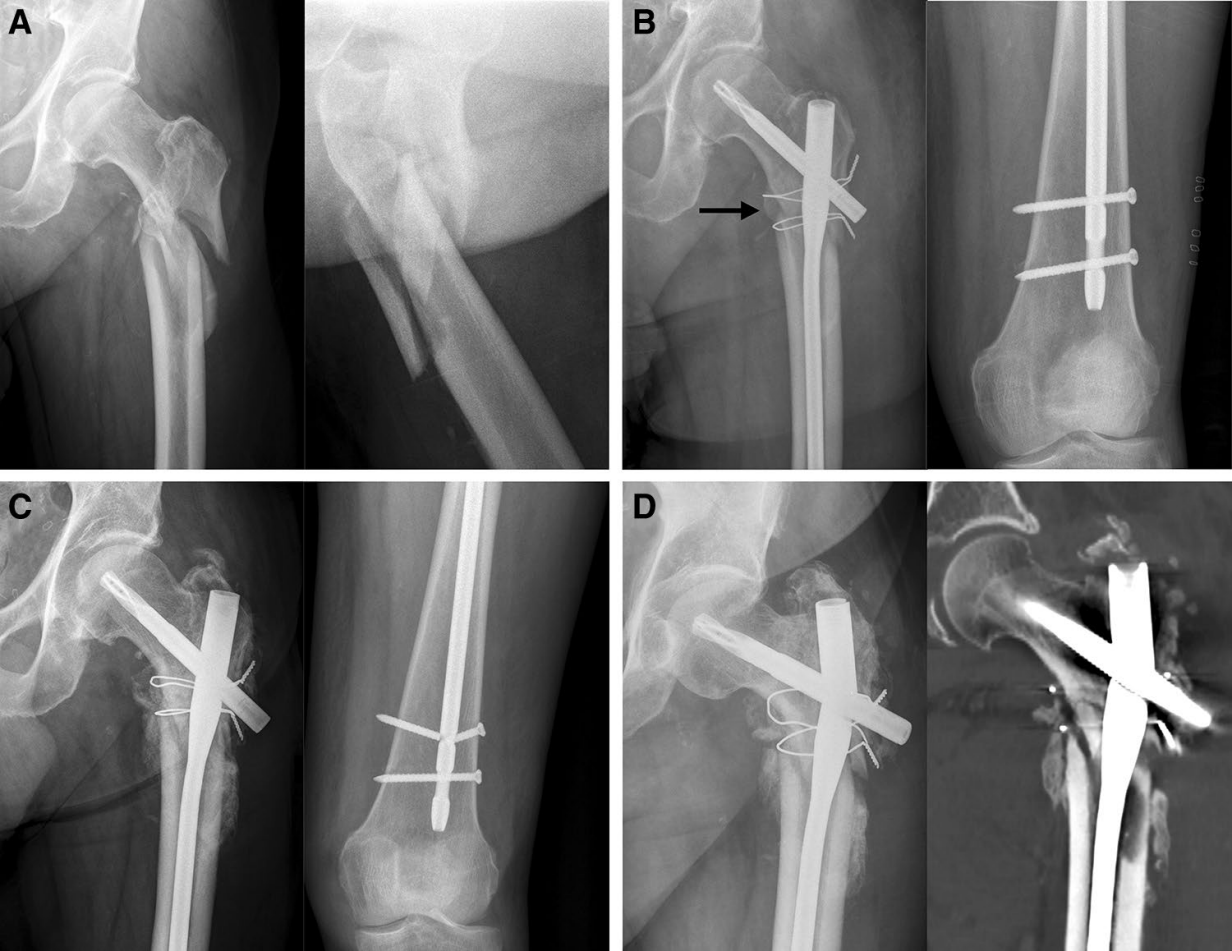
Seventy two-year-old male after a fall at home. **a** Radiographs obtained at admission showing a Seinsheimer Type IV fracture. **b** Postoperative radiographs after open reduction, cerclage wiring and intramedullary nailing: varus malalignment (as indicated by the displacement of the proximal medial cortex into the medullary canal with slight varus angulation relative to the distal medial cortex) and lack of medial cortical support due to nonanatomic reduction (black arrow), distal static and dynamic locking. **c** Unscheduled radiographs after 9 weeks due to persistent pain: no loss of reduction, timely callus formation, breakage of the static locking bolt and autodynamisation of the nail. There was no breakage of the static locking bolt at the previous routine controls. **d** Unscheduled radiograph (left) and CT scan (right) after 7 months due to suddenly increasing pain: nail breakage and no fracture healing

**Fig. 3:**
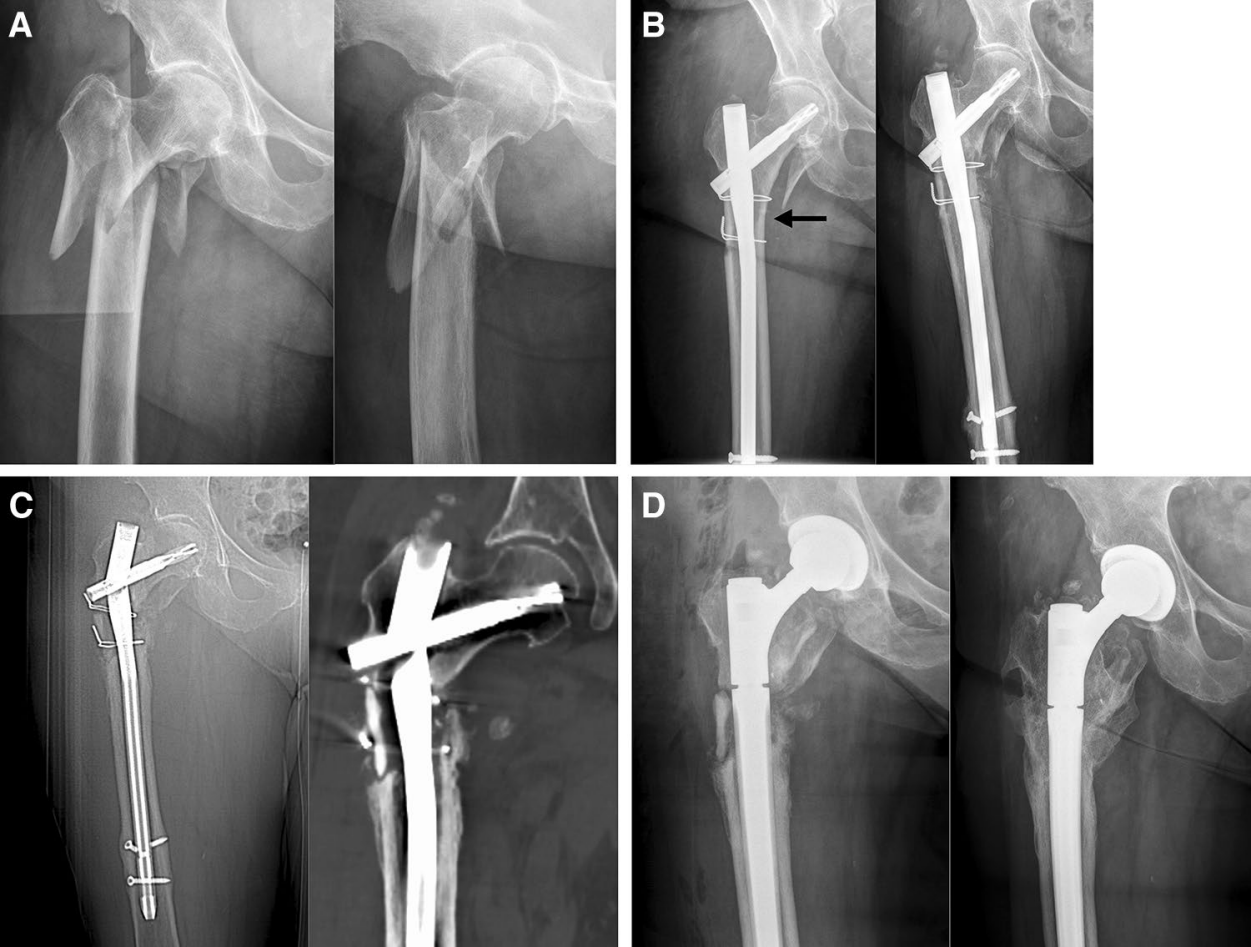
Eighty three-year-old female after a simple fall at home. **a** Radiographs obtained at admission showing a Seinsheimer Type V fracture. **b** Postoperative radiograph (left) after open reduction, cerclage wiring and intramedullary nailing: restoration of the subtrochanteric medial cortical support (black arrow) and no varus malalignment. The lesser trochanter fragment was not reduced. Scheduled radiograph after 12 weeks: no loss of reduction, timely callus formation, but breakage of the static locking bolt and autodynamisation of the nail. **c** Unscheduled radiograph (left) and CT scan (right) after 10 months due to suddenly increasing pain: nail breakage and no fracture healing. **d** Postoperative X-ray (left) after conversion to total hip arthroplasty using a modular revision stem anchored in the diaphyseal isthmus. The nonunion was not addressed surgically. Scheduled radiograph (right) 12 months after revision surgery: no component loosening and fracture healing

Osteoporosis (1c) was assessed using the cortical thickness index as described by Sah et al. [22] with values < 0.4 on lateral radiographs indicating osteoporosis. The determination as high- or low-energy trauma (2a) was based on the injury mechanism. The distance between the trochanter tip and the centre of the primary fracture line (2c) as well as varus malalignment (4a) and medial cortical support (4b) after reduction were measured on postoperative AP radiographs. Varus malalignment was defined as varus angulation of > 5 degrees. Figure 1 schematically shows the differences between “varus malalignment” (Fig. 1a), “no medial cortical support” (Fig. 1b), and “varus malalignment without medial cortical support” (Fig. 1c). As illustrated in Fig. 1b, postoperative lack of medial cortical support typically results from nonanatomic reduction, but can be also a consequence of medial comminution or a large displaced medial butterfly fragment. Residual translational fracture displacement after reduction was measured on postoperative AP (3a) and lateral radiographs (3b). The assessment of the proximal and distal locking of the nail (3c) included the presence or absence of a screw or blade in the femoral neck as well as static or dynamic locking distally. A static group (screw or blade in the femoral neck and static distal locking) was distinguished from a group with no implant in the femoral neck and/or dynamic distal locking. Autodynamisation (4c) was defined as breakage or loosening of distal locking screws within the first 12 weeks post-surgery (Figs. 2c, 3b) [13]. Implant failure, i.e., nail breakage, later than 6 months after primary surgery, was regarded as a result of and not as a risk factor for nonunion (Figs. 2d, 3c). Atypical fractures (5c) were defined as fractures after low-energy trauma or without history of trauma in patients with bisphosphonate intake for at least 1 year, lateral cortical thickening in the fracture zone and transverse, short oblique or z-shaped simple fractures. The presence of prodromal pain was optional.

SPSS 24 (IBM, Armonk, NY, USA) was used for statistical analysis. Metric data are reported as arithmetic mean ± standard deviation (SD), while categorical data are reported as absolute frequencies and percentage distribution. An independent samples *t* test or alternatively a non-parametric Mann–Whitney *U* test was used for the analysis of metric data depending on the distribution form. The distribution form was determined using the Kolmogorov–Smirnov test. A Chi-square test or a Fisher’s exact test for dichotomous variables were used for the analysis of categorical data. Additionally, the accuracy of each parameter for the development of nonunion was calculated. For this purpose, all nondichotomous parameters were further classified into two groups. For metric data (1a, 2c, 3a, 3b), the median value was used as a threshold for dichotomization. Fracture types (2b) were dichotomized in a group of two-part fractures (Seinsheimer type I and type II) and a group of three- or multipart fractures (Seinsheimer type III–V). The accuracy was calculated according to the following formula (see also Table 2):

$${\text{Accuracy}}=\frac{{{\text{True positive}}+{\text{true negative}}}}{{{\text{Total number of patients}}}}.$$

## Results

Between January 2012 and December 2017, 212 patients with the initial diagnosis of an acute subtrochanteric femoral fracture were treated at our level 1 trauma centre by femoral nailing. A review of the radiographs revealed that 91 fractures were either intertrochanteric fractures with subtrochanteric fracture extensions or proximal femoral shaft fractures and thus did not meet the strict definition of subtrochanteric fractures, which we applied for this study [9, 10, 19]. Of the remaining 121 patients, 47 were excluded from the study due to incomplete radiological follow-up, subsequent treatment in an external hospital or death during the follow-up period in geriatric patients. A total of 74 patients were therefore included in this study. There were 47 females and 27 males. Patients’ mean age was 70.2 ± 16.6 years (23–96 years).

Nonunion occurred in 17 of 74 patients (23.0%). There were 3 parameters with a significant effect on the nonunion rate (Fisher’s exact test, *p* < 0.05, Table 1): varus malalignment, lack of medial cortical support and autodynamisation of the nail. All other parameters (*n* = 12) did not have a significant effect on the nonunion rate. Autodynamisation of the nail occurred in 9 patients, of whom 8 developed nonunion (88.9%). All of these 9 nails were distally locked with two locking screws using a static and a dynamic locking option. Autodynamisation of the nail occurred by breakage of the static locking screws in all patients (Figs. 2c, 3b).

**Table 1:** Comparison of patients with and without nonunion after intramedullary nailing of subtrochanteric femoral fractures

		All patients	Nonunion	Union	*p* value
Age		70.2 ± 16.6	67.0 ± 15.9	71.2 ± 16.8	0.36
Gender	Male	27	7	20	0.78
	Female	47	10	37	
Osteoporosis (CTI lateral < 0.4)	Yes	50	10	40	0.39
	No	24	7	17	
Trauma	High-energy	17	5	12	0.52
	Low-energy	57	12	45	
Fracture type (Seinsheimer classification)	Type 1	2	0	2	0.59
	Type 2	33	5	28	
	Type 3	17	7	10	
	Type 4	7	2	5	
	Type 5	15	3	12	
Distance trochanter tip-fracture		92.2 ± 15.5	93.6 ± 16.0	91.8 ± 15.5	0.67
Residual displacement AP view		4.5 ± 4.7	4.3 ± 4.1	4.6 ± 4.9	0.81
Residual displacement lateral view		3.8 ± 4.6	3.2 ± 3.6	4.0 ± 4.8	0.50
Locking options	Static	68	15	53	0.62
	Dynamic	6	2	4	
Varus malalignment	Yes	22	9	13	0.03*
	No	52	8	44	
Restoration of the medial cortical support	Yes	44	5	39	0.01*
	No	30	12	18	
Autodynamisation	Yes	9	8	1	0.00*
	No	65	9	56	
Open reduction	Yes	48	14	34	0.15
	No	26	3	23	
Cerclage wires	Yes	36	9	27	0.79
	No	38	8	30	
Atypical fracture	Yes	11	2	9	0.99
	No	63	15	48	

Metric data are reported as arithmetic means ± standard deviations and categorical data as absolute frequencies

*p < 0.05

**Fig. 4:**
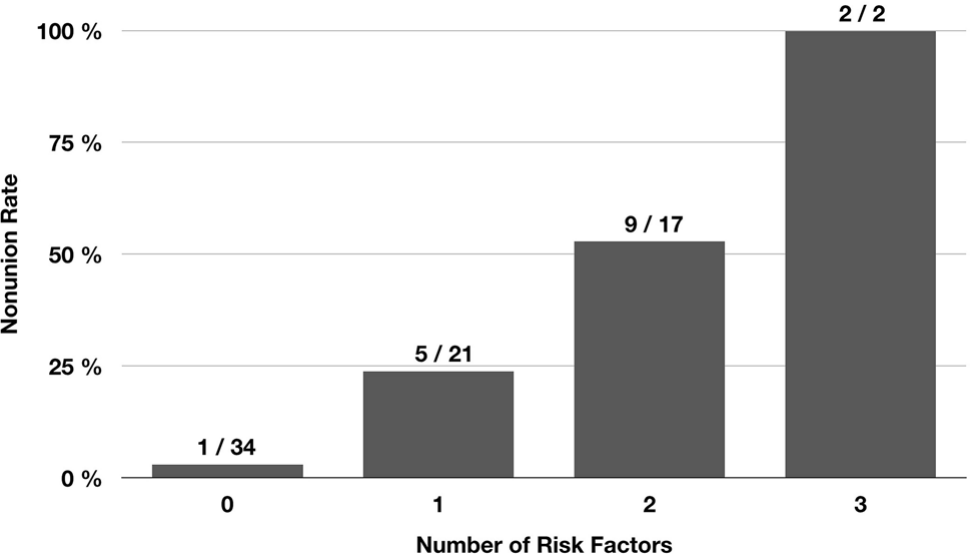
Effect of the number of risk factors on the nonunion rate. Autodynamisation, varus malalignment and lack of medial cortical support were defined as risk factors for nonunion. The nonunion rate significantly increased with the number of risk factors

Table 2 shows the accuracy of all parameters investigated to predict nonunion. The aforementioned three significant parameters also showed the highest accuracy with values > 0.70. The relatively high accuracy of the parameter “locking options” was attributed to the mismatch between the number of patients with static (> 90% of patients) and dynamic locking in our study (Table 1). The following three parameters were, therefore, defined as risk factors for nonunion after intramedullary nailing of subtrochanteric femoral fractures: varus malalignment, lack of medial cortical support and autodynamisation of the nail within the first 12 weeks post-surgery. Figure 4 shows that the nonunion rate significantly increased with the number of risk factors (Chi-square test, *p* < 0.001).

**Table 2:** Accuracy of the parameters analysed to predict nonunion

	Positive	Negative	Accuracy
Autodynamisation	Yes	No	0.86
Locking options	Dynamic	Static	0.74
Varus malalignment	Yes	No	0.72
Restoration of the medial cortical support	No	Yes	0.70
Trauma	High-Energy	Low-Energy	0.68
Atypical fracture	Yes	No	0.68
Gender	Male	Female	0.59
Fracture type (Sensheimer classification)	> Two-Part	Two-Part	0.57
Cerclage wires	Yes	No	0.53
Distance trochanter tip-fracture	> 90 mm	< 90 mm	0.51
Residual displacement AP view	> 2.7 mm	< 2.7 mm	0.51
Residual displacement lateral view	> 2.1 mm	< 2.1 mm	0.50
Open reduction	Yes	No	0.50
Age	> 73 years	≤ 73 years	0.49
Osteoporosis (CTI lateral < 0.4)	Yes	No	0.36

The accuracy of each parameter was calculated by dividing the sum of the number of ‘‘true positive’’ patients (positive parameter + nonunion) and ‘‘true negative’’ patients (negative parameter + no nonunion) by the total number of patients (*n* = 74)

## Discussion

Our data show that:

- varus malalignment, lack of medial cortical support and autodynamisation of the nail within the first 12 weeks post-surgery are significant risk factors for nonunion after intramedullary nailing of subtrochanteric femoral fractures.
- all significant risk factors are mechanical risk factors.
- the risk of nonunion considerably increases with the number of risk factors.
- none of the 12 patient-related, fracture-related, surgery-related and biological parameters included in our analysis did have a significant effect on the nonunion rate.

Intramedullary nailing of diaphyseal and metaphyseal fractures is one of the most successful techniques in orthopaedic trauma surgery [23]. It promotes secondary bone healing by providing relative stability and preserves the vascularity at the fracture site, especially the periosteal blood supply [24]. Accordingly, nonunion rates after intramedullary nailing are typically in a low single-digit percent range [25, 26]. Nonunion rates after intramedullary nailing of subtrochanteric femoral fractures, however, are markedly higher [13, 17, 19, 27, 28]. Distinct biological and mechanical properties of the subtrochanteric region may account for this finding. First, the subtrochanteric medial cortex is subject to massive bending forces with values as high as 8.2 MPa [7, 8, 29] under physiological loads [10, 15, 30]. These forces need to be neutralized by the implant until osseous healing has occurred. Second, the subtrochanteric region has been described as a region with poor vascularity and with a low blood flow rate by several authors [11–13, 19, 21]. It is well known that bone segments with distinct blood supply distribution are associated with higher nonunion rates. This applies, for example, for displaced femoral neck fractures, scaphoid fractures or Jones fractures of the fifth metatarsal bone. Santolini et al. [24], however, showed in an extensive review of multiple anatomical studies that the vascularity of the subtrochanteric region does not differ from other metaphyseal segments of long bones. Probably it is not the vascularity per se, but rather the very high bone turnover, which distinguishes the subtrochanteric region from other metaphyseal regions.

*Varus malalignment* (Figs. 1a, 2b) is a well-known risk factor for subtrochanteric nonunion [2, 3, 12–14, 18]. Varus malalignment results in increased bending forces on the medial subtrochanteric cortex. These forces need to be neutralized by the implant until osseous healing has occurred. On the one hand, intramedullary nails are therefore preferable to extramedullary devices due to shorter lever arms for the countertorque of bending moments [14]. On the other hand, intramedullary nailing per se is a risk factor for varus malalignment. The medullary canal in the inter- and subtrochanteric region is broad with a relatively short proximal fragment in subtrochanteric fractures facilitating varus malalignment [10, 17]. This is even more relevant for Seinsheimer Type V fractures with intertrochanteric fracture extension [9]. Choosing a correct nail entry point is thus of crucial importance [31] and individual patient anatomy must be taken into account. According to our own experience and consistent with the findings in the literature, we recommend the consideration of two aspects for the avoidance of varus malalignment. First, the fracture should be reduced prior to the insertion of the nail [13, 31, 32]. This facilitates identification of the correct nail entry point [10]. The nail should therefore not be used as a reduction tool for subtrochanteric fractures. Second, the ideal entry point must not be lateral to the trochanteric tip for proximally bent nails. In general, we recommend an entry point, which is located slightly medial to the trochanteric tip, for these nails to avoid varus malalignment. However, due to individual variations in the ideal trochanteric entry point, thorough preoperative analysis of the individual patient anatomy is essential [31, 33]. Alternatively, the use of straight nails with a piriformis fossa entry point (collinear with the long axis of the femoral shaft) may be a valuable option to decrease the risk of entry point-related malreduction [33, 34].

*Lack of medial cortical support* (Figs. 1b, 2b) is an additional previously described risk factor for nonunion after subtrochanteric fracture stabilisation [7, 10, 13, 21]. Medial cortical buttress after reduction supports the implant in the counteraction of bending forces and varus torque during postoperative mobilisation. While the intramedullary nail acts more like a load-sharing device in the presence of medial cortical support, it has to act more like a load-bearing device in the absence of medial cortical support [7, 15]. Anatomic reduction results in restoration of the medial cortical support, if there is a fracture without comminution zone. In comminuted fractures, however, this is not the case [21]. Although the problem of medial comminution is widely recognized, there are, to the best of the authors´ knowledge, no recommendations in the literature for this problem. In an analogical manner, lack of medial cortical support is the most frequent cause for varus failure after surgical fixation of proximal humerus fractures [35, 36]. Strategies to prevent varus failure at the proximal humerus include shortening by impaction of the shaft into the head fragment [35], fibular cortical allografts [37] or even “mushroom” tailored cancellous allografts [36, 38]. These options, however, may not be feasible and advisable for the subtrochanteric region. Giannoudis et al. [13] demonstrated a case of subtrochanteric fracture nonunion revised using a lateral 95 degree blade plate and an additional anterior plate. Double plating or nailing with an additional anteromedial buttress plate may be as well an option for acute subtrochanteric fractures with severe medial comminution zones.

*Autodynamisation of the nail* was found to be a strong risk factor for nonunion in our study, with 8 out of 9 patients with autodynamisation developing nonunion. In general, a risk factor for subtrochanteric nonunion is a variable associated with an increased risk of subtrochanteric nonunion, but this does not necessarily imply causality. It is debatable whether delayed union/nonunion and weakness of the mechanical construct causes autodynamisation or vice versa, particulary if autodynamisation occurs in an early phase postoperatively (“chicken-and-egg” dilemma). Giannoudis et al. [13] stated that autodynamisation should be considered as a consequence of rather than the cause for nonunion. In their study, autodynamisation occurred after a mean of 4.4 months with nail breakage following 2 months later on average (6.5 months). In our study, autodynamisation occurred after 12 weeks at the latest and therefore much earlier, whereas nail breakage happened between 6 and 12 months after the index surgery resulting in a much longer interval between autodynamisation and nail breakage. One reason for these differences may be the inclusion of patients with atrophic nonunions only in the study by Giannoudis et al. [13]. We agree with the authors that autodynamisation is an indicator for instability of the overall mechanical construct and that it is predictive of future nonunion and nail breakage [13]. The long time period between autodynamisation and nail breakage, however, provides an opportunity for therapeutical interventions. Furthermore, Giannoudis et al. recommended revision surgery or weight-bearing restrictions, if autodynamisation is observed in patients who are still symptomatic at the fracture level. According to our experience, weight-bearing restrictions should not be supposed to be the solution in these cases. We therefore rather recommend revision surgery, if autodynamisation of the nail is observed in the early postoperative period. Accordingly, dynamisation of the nail by removing static locking screws, in our opinion, is absolutely contraindicated after intramedullary nailing of subtrochanteric fractures.

One of the main findings of our study is that all three risk factors for subtrochanteric nonunion were mechanical parameters. Another interesting, but not surprising, finding is that the nonunion rate increased with the number of risk factors (Fig. 4). The identified risk factors either result in increased load on the implant (varus malalignment), reduced intrinsic stability of the fracture (lack of medial cortical support) or reduced stability of the overall mechanical construct (autodynamisation). It is, therefore, reasonable to assume that there is a summation effect.

In contrast to the mechanical parameters, we did not find significant biological risk factors for nonunion in our study. Our subjective observations confirm this finding. First, we found timely callus formation in all nonunion cases (Figs. 2c, 3b) indicating a favourable biological environment for bone healing. Second, breakage of the nail was the characteristic failure mode, which indicates an unfavourable mechanical environment. Additionally, Fig. 3d shows osseous healing of a nonunion following nail removal and implantation of a modular revision stem without addressing the nonunion surgically at all. Our findings are in line with those reported by other authors. Although open reduction was defined as a risk factor for subtrochanteric nonunion by some authors [19, 39], there is increasing evidence that the mechanical advantages of preventing varus malalignment and restoration of the medial cortical support outweigh the biological disadvantages of open reduction [8, 10, 29]. Closed reduction is, therefore, advisable only, if it does not forfeit these mechanical advantages [12]. The same applies for the use of cerclage wires. The periostal vascular supply in the subtrochanteric region is circumferential [2, 11, 14, 19]. It has been shown that the vascular supply is preserved after using cerclage wires [2, 32, 40]. We therefore consider the application of subtrochanteric cerclage wires to be safe and valuable, if they facilitate anatomic fracture reduction and stabilization [10, 21]. Cerclage wiring also appears to be useful to reduce displaced large medial butterfly fragments (Fig. 1b, *middle*) to restore medial cortical support and to improve medial cortex healing. Furthermore, subtrochanteric fractures after long-term treatment with bisphosphonates have been described as prone to nonunion. Bisphosphonates inhibit osteoclastic bone remodelling and therefore lengthen the transformation of calcified callus to mature bone tissue [20, 41]. Indeed, several studies showed delayed bone union in these cases, but the nonunion rate was not increased [3, 4]. From a mechanical point of view, it is relatively simple to restore the medial cortical support using intramedullary nails, since these fractures are typically transverse fractures without comminution zones. Accordingly, prevention of varus malalignment represents the major surgical challenge in the surgical treatment of atypical subtrochanteric fractures.

Several limitations of our study should be noted. First, this is a retrospective study with all limitations associated with this study design. Second, increasing the number of included patients would have increased the power of the statistical analysis. Third, potential risk factors, such as smoking, diabetes and medication intake, were not assessed, as this study primarily focused on potential risk factors that may be influenced by the surgeon (e.g., postoperative reduction and alignment, open vs. closed reduction). Fourth, and as expected, the mean age within this consecutive series of patients was relatively high (70.2 ± 16.6 years, range 23–96 years) and about three quarters of fractures resulted from low-energy trauma (Table 1). As a consequence, the findings of this study might not be generalisable to high-energy subtrochanteric fractures in young patients. Fifth, X-rays were used for the measurement of angulation and displacement as well as for the assessment of osteoporosis. Finally, clinical outcome as well as revision strategies for the nonunion cases were beyond the topic of this manuscript.

In conclusion, the results of this retrospective study indicate that prevention of postoperative varus malalignment and restoration of the medial cortical support are the most critical factors to prevent nonunion after intramedullary nailing of subtrochanteric femoral fractures. Moreover, autodynamisation of the nail in the early postoperative period was found to be a strong predictor for failure and thus should result in revision surgery. All of these three significant risk factors identified are mechanical risk factors. In contrast, none of the investigated biological parameters, such as open reduction, did have a significant effect on the nonunion rate in this patient series. Careful soft tissue dissection, however, is deemed mandatory during open reduction.

## References

[1] Ekström W, Németh G, Samnegård E, Dalen N, Tidermark J (2009). Quality of life after a subtrochanteric fracture: a prospective cohort study on 87 elderly patients. Injury.

[2] Codesido P, Mejía A, Riego J, Ojeda-Thies C (2017). Subtrochanteric fractures in elderly people treated with intramedullary fixation: quality of life and complications following open reduction and cerclage wiring versus closed reduction. Arch Orthop Trauma Surg.

[3] Egol KA, Park JH, Rosenberg ZS, Peck V, Tejwani NC (2014). Healing delayed but generally reliable after bisphosphonate-associated complete femur fractures treated with IM nails. Clin Orthop Relat Res.

[4] Bogdan Y, Tornetta P, Einhorn TA, Guy P, Leveille L, Robinson J (2016). Healing time and complications in operatively treated atypical femur fractures associated with bisphosphonate use: a multicenter retrospective cohort. J Orthop Trauma.

[5] Kim KK, Won Y, Smith DH, Lee GS, Lee HY (2017). Clinical results of complex subtrochanteric femoral fractures with long cephalomedullary hip nail. Hip Pelvis.

[6] Matre K, Havelin LI, Gjertsen JE, Vinje T, Espehaug B, Fevang JM (2013). Sliding hip screw versus IM nail in reverse oblique trochanteric and subtrochanteric fractures. A study of 2716 patients in the Norwegian Hip Fracture Register. Injury.

[7] Celebi L, Can M, Muratli HH, Yagmurlu MF, Yuksel HY, Biçimoğlu A (2006). Indirect reduction and biological internal fixation of comminuted subtrochanteric fractures of the femur. Injury.

[8] Saini P, Kumar R, Shekhawat V, Joshi N, Bansal M, Kumar S (2013). Biological fixation of comminuted subtrochanteric fractures with proximal femur locking compression plate. Injury.

[9] Loizou CL, McNamara I, Ahmed K, Pryor GA, Parker MJ (2010). Classification of subtrochanteric femoral fractures. Injury.

[10] Beingessner DM, Scolaro JA, Orec RJ, Nork SE, Barei DP (2013). Open reduction and intramedullary stabilisation of subtrochanteric femur fractures: a retrospective study of 56 cases. Injury.

[11] Kim JW, Park KC, Oh JK, Oh CW, Yoon YC, Chang HW (2014). Percutaneous cerclage wiring followed by intramedullary nailing for subtrochanteric femoral fractures: a technical note with clinical results. Arch Orthop Trauma Surg.

[12] Riehl JT, Koval KJ, Langford JR, Munro MW, Kupiszewski SJ, Haidukewych GJ (2014). Intramedullary nailing of subtrochanteric fractures—does malreduction matter?. Bull Hosp Jt Dis.

[13] Giannoudis PV, Ahmad MA, Mineo GV, Tosounidis TI, Calori GM, Kanakaris NK (2013). Subtrochanteric fracture non-unions with implant failure managed with the “Diamond” concept. Injury.

[14] Panteli M, Mauffrey C, Giannoudis PV (2017). Subtrochanteric fractures: Issues and challenges. Injury.

[15] Li F, Sang W, Wang Q, Huang J, Lu H (2011). Subtrochanteric fracture treatment: a retrospective study of 46 patients. Med Princ Pract.

[16] von Rüden C, Hungerer S, Augat P, Trapp O, Bühren V, Hierholzer C (2015). Breakage of cephalomedullary nailing in operative treatment of trochanteric and subtrochanteric femoral fractures. Arch Orthop Trauma Surg.

[17] Park SH, Kong GM, Ha BH, Park JH, Kim KH (2016). Nonunion of subtrochanteric fractures: comminution or malreduction. Pak J Med Sci.

[18] Shukla S, Johnston P, Ahmad MA, Wynn-Jones H, Patel AD, Walton NP (2007). Outcome of traumatic subtrochanteric femoral fractures fixed using cephalo-medullary nails. Injury.

[19] Hoskins W, Bingham R, Joseph S, Liew D, Love D, Bucknill A (2015). Subtrochanteric fracture: the effect of cerclage wire on fracture reduction and outcome. Injury.

[20] Kharwadkar N, Mayne B, Lawrence JE, Khanduja V (2017). Bisphosphonates and atypical subtrochanteric fractures of the femur. Bone Joint Res.

[21] Choi JY, Sung YB, Yoo JH, Chung SJ (2014). Factors affecting time to bony union of femoral subtrochanteric fractures treated with intramedullary devices. Hip Pelvis.

[22] Sah AP, Thornhill TS, LeBoff MS, Glowacki J (2007). Correlation of plain radiographic indices of the hip with quantitative bone mineral density. Osteoporos Int.

[23] Bong MR, Kummer FJ, Koval KJ, Egol KA (2007). Intramedullary nailing of the lower extremity: biomechanics and biology. J Am Acad Orthop Surg.

[24] Santolini E, Goumenos SD, Giannoudi M, Sanguineti F, Stella M, Giannoudis PV (2014). Femoral and tibial blood supply: a trigger for non-union?. Injury.

[25] Canadian Orthopaedic Trauma Society (2003). Nonunion following intramedullary nailing of the femur with and without reaming. Results of a multicenter randomized clinical trial. J Bone Jt Surg Am.

[26] Rupp M, Biehl C, Budak M, Thormann U, Heiss C, Alt V (2018). Diaphyseal long bone nonunions—types, aetiology, economics and treatment recommendations. Int Orthop.

[27] Barquet A, Mayora G, Fregeiro J, Lopez L, Rienzi D, Francescoli L (2004). The treatment of subtrochanteric nonunions with the long gamma nail: twenty-six patients with a minimum 2-year follow-up. J Orthop Trauma.

[28] Haidukewych GJ, Berry DJ (2004) Nonunion of fractures of the subtrochanteric region of the femur. Clin Orthop Relat Res (419):185–188 10.1097/00003086-200402000-00030 15021152

[29] Lourenço PR, Pires RE, Barbosa de Toledo (2016). Subtrochanteric fractures of the femur: update. Rev Bras Ortop.

[30] Saarenpää I, Heikkinen T, Jalovaara P (2007). Treatment of subtrochanteric fractures. A comparison of the Gamma nail and the dynamic hip screw: short-term outcome in 58 patients. Int Orthop.

[31] Ostrum RF, Marcantonio A, Marburger R (2005). A critical analysis of the eccentric starting point for trochanteric intramedullary femoral nailing. J Orthop Trauma.

[32] Mingo-Robinet J, Torres-Torres M, Moreno-Barrero M, Alonso JA, García-González S (2015). Minimally invasive clamp-assisted reduction and cephalomedullary nailing without cerclage cables for subtrochanteric femur fractures in the elderly: Surgical technique and results. Injury.

[33] Streubel PN, Wong AH, Ricci WM, Gardner MJ (2011). Is there a standard trochanteric entry site for nailing of subtrochanteric femur fractures?. J Orthop Trauma.

[34] Ricci WM, Gallagher B, Haidukewych GJ (2009). Intramedullary nailing of femoral shaft fractures: current concepts. J Am Acad Orthop Surg.

[35] Krappinger D, Bizzotto N, Riedmann S, Kammerlander C, Hengg C, Kralinger FS (2011). Predicting failure after surgical fixation of proximal humerus fractures. Injury.

[36] Euler SA, Hengg C, Wambacher M, Spiegl UJ, Kralinger F (2015). Allogenic bone grafting for augmentation in two-part proximal humeral fracture fixation in a high-risk patient population. Arch Orthop Trauma Surg.

[37] Matassi F, Angeloni R, Carulli C, Civinini R, Di Bella L, Redl B (2012). Locking plate and fibular allograft augmentation in unstable fractures of proximal humerus. Injury.

[38] Euler SA, Kralinger FS, Hengg C, Wambacher M, Blauth M (2016). Allograft augmentation in proximal humerus fractures. Oper Orthop Traumatol.

[39] Malik MH, Harwood P, Diggle P, Khan SA (2004). Factors affecting rates of infection and nonunion in intramedullary nailing. J Bone Jt Surg Br.

[40] Apivatthakakul T, Phaliphot J, Leuvitoonvechkit S (2013). Percutaneous cerclage wiring, does it disrupt femoral blood supply? A cadaveric injection study. Injury.

[41] Rizzoli R, Åkesson K, Bouxsein M, Kanis JA, Napoli N, Papapoulos S (2011). Subtrochanteric fractures after long-term treatment with bisphosphonates: a European society on clinical and economic aspects of osteoporosis and osteoarthritis, and international osteoporosis foundation working group report. Osteoporos Int.

